# Antihyperglycemic and Antihyperlipidemic Effects of Fruit Aqueous Extract of Berberis integerrima Bge. in Streptozotocin-induced Diabetic Rats

**Published:** 2014

**Authors:** Hossein Ashraf, Reza Heidari, Vahid Nejati

**Affiliations:** *Department of Biology, Faculty of Science, Urmia University, Urmia, Iran.*

**Keywords:** *Berberisintegerrima*, *Diabetesmellitus*, *Hyperglycemia*, *Hyperlipidemia*, *Rat*

## Abstract

Use of medicinal plants for attenuation of hyperglycemia and restoration of lipids disorder to normal level is clinically very important. The aim of present study was to evaluate the effects of *Berberis integerrima *Bge. fruit aqueous extract (BIFAE) on blood glucose and lipid profile in streptozotocin (STZ) - induced diabetic rats*. *The STZ-induced diabetic rats were treated by fruit aqueous extract of *Berberis integerrima Bge. *at doses (250 and 500 mg/Kg bw) and glibenclamide (0.6 mg/Kg bw) for 42 days by gavage. Blood glucose levels and body weights of rats were measured on weeks 0, 2, 4 and 6. Total lipid levels were determined in normal and STZ-induced diabetic rats after administration of the BIFAE and glibenclamide for 42 days. STZ-induced diabetic rats showed a significant (P<0.001) increases in the levels of blood glucose, triglycerides (TG), total cholesterol (TC), low density lipoprotein LDL-cholesterol (LDL-C) while body weight and high density lipoprotein HDL-cholesterolan (HDL-C) were significantly(P<0.001) decreased compared to normal rats. Daily administration of BIFAE did not possess the hypoglycemic and hypolipidaemic activity in STZ- diabetic rats during 6-week treatment period. Results indicate the usage of BIFAE in traditional medicine for the treatment of diabetes may need more investigation.

## Introduction

Use of medicinal plants in traditional medicine to treat diabetes has a long history, although there are few experiences and scientific results in scientific societies (1). Diabetes mellitus is a chronic metabolic disorder, lifetime and one of the most common endocrine diseases. In most cases, it is due to reduced insulin secretion by beta cells of pancreatic islets (2).This disease is characterized by metabolic disorders, long-term complications on the eyes, kidneys, nerves and blood vessels and it entails a wide disturbance in the metabolism of carbohydrates, fats, proteins, water and electrolytes (3). Due to numerous injuries and occasionally fatal disease caused in diabetic patients, there is a need to find the treatment methods and ways to reduce and prevent it. At the present time, with regard to the problems of preparing and injecting insulin and other glucose-lowering drugs and also considering the side effects of synthetic drugs, the use of herbal medicines has attracted the attention of researchers.*Berberis integrrima *Bge. (Syn: *Berberis* densiflora Boiss.&Buhse) is a medicinal shrub with yellow wood and obovate leaves, bearing pendulous yellow flowers succeeded by oblong red fruits. This plant belongs to the *Berberidaceae* and found in most regions of Iran, especially in northern and northeastern.

Due to having secondary metabolites such as Berberine, Oxyacanthine, Bermamine, Palmatine, Jateorrhizine, Columbamineand Berberubine, this plant has too much medicinal properties (4, 26).Various properties are listed for different parts of barberry plant and these properties have been confirmed in various research (5-7). In addition to the antioxidant properties of Barberry fruit (5), a variety of alkaloids obtained from root and stem bark, which most important of them is Berberine (6). Based on studies on barberry root extract and its main alkaloid (Berberine) these following properties are listed: Antioxidants (5), anti-inflammatory effects (6), hypoglycemia (7), hypolipidemic (8),^.^collecting free radicals and finally reduction of oxidative stress (9). However most of these studies performed in animal models and used of berberine (root and stem extract of barberry). Given that barberry fruits contain polyphenols, pectin and gum, vitamin C and malic acid (10), the present study was designed to evaluate the effects of *Berberis integerrima *Bge. fruit aqueous extract on blood glucose and lipid profile in diabetic rats. Also these effects was compared with standard drug, glibenclamide (0.6 mg/Kg bw). 

## Experimental

*Chemicals*

Streptozotocin (STZ) was purchased from Sigma Chemical Co. (St. Louis, MO, USA). Total cholesterol, triglyceride, high-density lipoprotein standard kits were obtained from Pars Azmoon, Tehran, Iran. All reagents used in study were of analytical grade.

*Plant material*

Wild samples of Barberry fruit (Berber isintegrrima Bge.) were collected from suburb Bavanat City (Fars Province, Iran) during November and December 2011 and identified by the Botany Department of Urmia University. A voucher specimen of the plant was deposited in the herbarium of the Faculty of Sciences, Urmia University, Urmia, Iran (No. 9059). 

*Preparation of aqueous extract*

Fruits were dried in the shade and finely powdered. The aqueous extract was prepared by cold maceration of 150 g of powdered fruit in 500 mL of distilled water for 72 h. Then the extract was filtered through a Whatman No.1 filter paper to obtain a clear extract. The filtrate was concentrated by water bath (65 °C) for 48 h, dried in vacuum (yield 10 g) and the residue was stored in a refrigerator at 2-8 °C for use in subsequent experiments (11). The required concentration was prepared in accordance mg/Kg body weight by normal saline.

*Animals*

Male wistar rats, weighing about 180-220 g (obtained from the central animal house of the Tehran Pasteur Institute, Tehran, Iran) were used in the study. Animals were maintained under standard environmental condition, i.e. ambient temperature of 22 ± 2 °C and at 45-55% relative humidity, 12 h each of dark and light cycle and fed with a standard pellet rats diet ad libitum. Water was supplied ad libitum. All the studies were conducted in accordance with the Animal Ethical Committee of the University.

*Acute toxicity study*

Acute toxicity study of aqueous extract of *Berberis integerrima *Bge. was determined as per the OECD guideline No. 423 (Acute Toxic Class Method). It was observed that test extract was not lethal to the rats even at 2500 mg/Kg dose. Hence, 10% (250 mg/Kg) and 20% (500 mg/Kg) of this dose were selected for further study (12).

Experimental induction of diabetes

Diabetes was induced in rats by intraperitoneal injection of streptozotocin (STZ) at a dose of 65 mg/Kg bw, dissolved in 0.1 cold citrate buffer [ph=4.5] (13). Blood samples were taken from the tail vein 72 h after STZ injection to measure blood glucose levels by ACCU-Check glucose meter. Only animals with fasting blood glucose levels (after fasting for 12 hours) over 300 mg/dl were considered diabetic and used for the further studies (14).

*Experimental design*

All animals were randomly divided into five groups with six animals in each group.

Group I. Normal control treated with normal saline (10 mL/Kg).

Group II. Diabetic control treated with normal salin (10 mL/Kg).

Group III. Diabetic rats treated aqueous extract of Berberis integrrima Bge. fruit (250 mg/Kg body weight).

Group IV. Diabetic rats treated aqueous extract of Berberis integrrima Bge. fruit (500 mg/Kg body weight).

Group V. Diabetic rats treated with Glibenclamide (0.6 mg/Kg of body weight)(15).

Animals were treated daily by gavage for 6 weeks. The experimental periods for each rat were 6 weeks.

At the end of the study, animals were fasted overnight and anesthetized with chloroform (Pharmaceutical Partners of Japan). Blood samples were collected from the animal's heart and the serum was separated by centrifugation (3000 rpm at 40 °C for 15 min) and stored at -30 °C for different biochemical analyses.

*Estimation of body weight*

The body weights in experimental animals were determined before the study, and at 2, 4 and 6 weeks after the study by a digital balance. These weights were determined at the same time during the morning.

*Estimation* *of blood glucose*

Throughout the 6-week treatment period, fasting (12 hours) blood glucose was measured before the study, and at 2, 4 and 6 weeks after the study on lateral tail vein. blood samples were analyzed using an ACCU-Check glucose meter (Roche, Mannheim, Germany).

Estimation of blood lipid profile

*Lipid in serum concentration including *triglycerides, total cholesterol, high density lipoprotein HDL-cholesterol (HDL-C) determined with the use of commercially available enzymatic kits (Pars Azmoon, Theran, Iran) and using an automatic analyzer (Architect c8000 Clinical Chemistry System, USA). LDL cholesterol (LDLC) was estimated by Frydvald method:

 LDLcholsterol = total cholesterol- HDL cholesterol- (Triglyceride ÷ 5).

*Statistical analysis*

All the data reported are expressed as mean ± S.E.M. Statistical analysis was performed by using one‐way ANOVA followed by Tukey’s multiple tests using 18 version of computer software. The values were considered statistically significant when p-value <0.05 compared to respective control.

## Results

*Effect on body weight of rats*

There was continuous reduction in body weight in diabetic rats. Glibenclamide significantly (P<001) improved the body weight of diabetic rats. Treatment of diabetic rats for 6 weeks with the aqueous extract of barberry fruit did not change the body weight in comparison to untreated diabetic rats (Table 1).

**Table 1:** Effect of glibenclamide and BIFAE on Average body weight in normal and diabetic rats

**Group** **(n=6)**	**Treatment**	**Dose** **(mg/Kg)**	**Average body weight (g)**			
			**Week0**	**Week2**	**Week4**	**Week6**
1	**N+C**	10 mL/Kg	193.20±6.58	211.56±4.74	233.34±3.20^a^	247.62±1.87^a^
2	**D+C**	10 mL/Kg	206.84±3.35	155.28±4.97#^a^	144.90±2.01#^a^	137.98±2.24#^a^
3	**D+BIFAE**	250	191.88±4.54	169.10±3.32^a^	148.52±5.78^a^	140.48±1.18^a^
4	**D+BIFAE**	500	196.62±4.17	167.8±4.66^a^	147.12±2.02^a^	145.08±2.00^a^
5	**D+G**	0.6	199.94±1.72	184.30±4.08*	193.46±5.53*	203.52±5.61*

**BIFAE:**fruit aqueous extract of *Berberis integerrima *Bge.; N normal;Ccontrol;Gglibenclamide; D diabetic; Values are presented as mean ± S.E.M..; n = 6 in each group. One way ANOVA followed by tukeytest.

# p<0.001 Diabetic control rats were compared with Normal control Rats.

* p<0.001 Diabetic treated rats were compared with diabetic control rats on corrspondingday;

a p<0.001 compared to 0 value.

*Antihyperglycemic activity*

Serum glucosemeasurements indicated that before diabetes induction, there were no significant differences among animals in each group. Single dose streptozotocin significantly increased the blood glucose. After the daily administration of glibenclamide*(0.6 mg/Kg bw)*for 42 days, significant decreases (P<0.001) in the blood glucose levels were observed in the diabetic rats , but treatment of diabetic rats for 6 weeks with the aqueous extract of barberry fruit did not change the serum glucose concentration in comparison to untreated diabetic rats (Table 2).

**Table 2:** Effect of glibenclamide and BIFAE on the blood glucose in normal and diabetic rats

**Group** **(n=6)**	**Treatment**	**Dose** **(mg/Kg)**	**Blood glucose level (mg/dl)**			
			**Week 0**	**Week 2**	**Week 4**	**Week 6**
1	**N+C**	10 mL/Kg	89.40 ± 5.98	89.20 ± 4.50	98.20 ± 4.52	93.80 ± 3.35
2	**D+C**	10 mL/Kg	89.40 ± 4.73	316.00 ± 2.16#^a^	327.00 ± 3.92#^a^	337.20 ± 7.53#^a^
3	**D+BIFAE**	250	94.2 ± 2.47	303.4 ± 2.33^a^	294.19 ± 19.08^a^	325.08 ± 6.4^a^
4	**D+BIFAE**	500	86.4 ± 6.51	307.4 ± 6.16^a^	312.4 ± 6.36^a^	330.4 ± 5.35^a^
5	**D+G**	0.6	87.20 ± 6.41	186.40 ± 2.37a *	147.40 ± 3.60a*	113.00 ± 3.43^a^ *

**BIFAE:**fruit aqueous extract of *Berberis integerrima *Bge.; N normal;Ccontrol;Gglibenclamide; D diabetic; Values are presented as mean ± S.E.M..; n = 6 in each group. One way ANOVA followed by tukeytest.

# p<0.001 Diabetic control rats were compared with normal control rats.

* p<0.001 Diabetic treated rats were compared with diabetic control rats on corrsponding day;

a p<0.001 compared to 0 value.

*Antihyperlipidemic effects*

Diabetes is also associated with altered lipid profile. There was a significant increase (P<0.001) in serum total cholesterol, triglycerides, LDL-cholesterol, and significant decrease (P<0.001) in HDL-cholesterol in diabetic rats compared to that of normal control. Glibenclamide cased a significantly decrease (P<0.001) in levels of total cholesterol, LDL-cholesterol, triglycerides and a significantly rise in HDL cholesterol level in comparison to untreated diabetic rats. Meanwhile, comparing barberry fruit-treated and untreated diabetic groups showed that there was no difference between this groups after 6 weeks regarding serum total cholesterol, HDL- cholesterol, LDL-cholesterol and triglycerides concentrations **(**Table 3**). **

**Table 3:** Effect of glibenclamide and BIFAE on Serum lipid profiles in normal and diabetic rats

**Group** **(n=6)**	**Treatment**	**Dose** **(mg/Kg)**	**Serum lipid profiles (mg/dl)**			
			**TC**	**TG**	**HDL**	**LDL**
1	**N+C**	10 mL/Kg	76.28 ± .98	66.42 ± 1.03	38.54 ± .48	24.25 ± 1.21
2	**D+C**	10 mL/Kg	125.94 ± 1.67#	121.68 ± .90#	14.46 ± .32#	87.14 ± 1.63#
3	**D+BIFAE**	250	121.76 ± 2.28	124.46 ± 2.33	294.19 ± 19.08	14.84 ± 0.32
						
4	**D+BIFAE**	500	119.96 ± 3.51	118.23 ± 6.16	312.4 ± 6.36	15.72 ± 0.45
5	**D+G**	0.6	97.70 ± 2.49*	81.53 ± .94*	35.20 ± .56*	46.18 ±2.67*

**BIFAE:**fruit aqueous extract of *Berberis integerrima *Bge. ; N normal;Ccontrol;Gglibenclamide; D diabetic; TC total cholesterol; TG triglyceride. Values are presented as mean ± S.E.M..; n = 6 in each group. One way ANOVA followed by tukeytest.

# p<0.001 Diabetic control Rats were compared with Normal control Rats.

* p<0.001 Diabetic treated rats were compared with diabetic control rats.

## Discussion

In the present study the hypoglycemic and hypolipidemic activity of fruit aqueous extract of *Berberis integerrima *Bge. was evaluated in streptozotocin induced diabetic rats. It is well known that injection of a high dose of STZ (>45 mg/Kg) significantly damages the ability of pancreatic β-cell to synthesize and secrete insulin in rats (16). Consequently, these animals develop impaired insulin response to food ingestion and glucose loading, and accordingly, impaired glucose uptake/utilization capabilities(17, 18)mimicking human type 1 diabetes mellitus. In our study, we have noticed significantly increased levels of serum glucose, total cholesterol, triglycerides and LDL- cholesterol but markedly decreased level of serum HDL- cholesterol in STZ- induced diabetic rats. The main cause of STZ-induced β-cell death is alkylation of DNA by the nitrosourea moiety of this compound. However, production of NO and reactive oxygen species may also be involved in DNA fragmentation and other deleterious effects of STZ (16).

The present study showed the glibenclamide at a dose of 0.6 mg/Kg bw had favourably modified serum glucose, body weight and lipid profile in rats with significant decreasesin serum glucose, total cholesterol, LDL-cholesterol, triglycerides and increased the body weight and serum HDL-C level in diabetic rats. These findings are consistent with earlier report showed that administration of glibenclamide at a dose of 0.6 mg/Kg bw to diabetic rats caused a significant decrease in blood glucose and total lipids in comparison to normal control *(*15). Our present data also showed that the aqueous extract of *Berberis integerrima *Bge. fruit at amount of 250 and 500 mg/Kg bw did not possess the hypoglycemic and hypolipidemic activity in STZ-diabetic rats during the 6-week treatment period. This finding are consistent with earlier report that showed aqueous extract of *Berberis vulgaris* fruit at doses 3.5 and 7.5% in drinking water did not have hypoglycemic and hypolipidemic activity in STZ-diabetic rats (19). Also incontrast with earlier report that showed a significant decreases in serum TG, TC, LDL-c, apo B, glucose and insulin at the end of the study in *Berberis vulgaris *fruit exract (3 g/d for 3 months) group in comparison to the control one (20). Therefore possess the hypolipidemic activity of *Berberis* *vulgaris* fruit in STZ-diabetic rats in these studies and not possess the hypolipidemic activity of *Berberis integerrima *Bge. fruit in our finding may dependent to amount of dose and time consumable. Because in our study the lipid profile decreased in diabetic rats treated by BIFAE specially at dose 500 mg/Kg bw, but this alteration was not significant.

Another reason for the results is that barberry fruit may contain low berberine, although the barberry root contain high berberine. hypoglycemic and hypolipidemic effects of berberine has been proved in many studies (7,8,21,22). Berberine may act as a *α*-glucosidase inhibitor, which is its main mechanism in diabetes treatment. The main mechanism of berberine in diabetes treatment may be act as an the *α*-glucosidase inhibitor. The inhibitory effect of berberine on diabetes also might be associated with its hypoglycemic effect, modulating lipids metabolic effects and its ability to scavenge free radicals (23). However, the inhibition of intestinal glucose absorption or stimulation of peripheral glucose uptake also could be the another mechanisms of hypoglycemic effect of berberine. Increased AMP-activated protein kinase (AMPK) activity in 3T3-L1 adipocytes and L6 myotubes and reduced the lipid accumulation in T3-L1 adipocytes resulted of berberine treatment (24). On the other hand, inhibition of mitochodrial function (inhibited oxygen consumption, increase AMP/ATP ratio and AMPK activation) by berberine can lead to up-regulation of glucose and lipid metabolism (25).

## Conclusion

This study showed that the fruit extract of *Berberis integerrima *Bge*. *amount of 250 and 500 mg/Kg bw did not possess the hypoglycemic and hypolipidemic activity in STZ-diabetic rats. Therefore, the usage of barberry fruit in traditional medicine for the treatment of diabetes may need more investigate
